# Sleep duration and problem behaviour in 8-year-old children in the Childhood Obesity Project

**DOI:** 10.1007/s00787-021-01731-8

**Published:** 2021-02-24

**Authors:** Kathrin Guerlich, Dariusz Gruszfeld, Justyna Czech-Kowalska, Natàlia Ferré, Ricardo Closa-Monasterolo, Françoise Martin, Pascale Poncelet, Elvira Verduci, Berthold Koletzko, Veit Grote

**Affiliations:** 1 Division of Metabolic and Nutritional Medicine, Department of Pediatrics, Dr. von Hauner Children’s Hospital, LMU University Hospital Munich, Lindwurmstr. 4, 80337 Munich, Germany; 2 Neonatal Intensive Care Unit, Children’s Memorial Health Institute, Warsaw, Poland; 3 Paediatrics Research Unit, Universitat Rovira i Virgili, IISPV, Reus, Spain; 4 Centre Hospitalier Chretien St. Vincent, Liège-Rocourt, Belgium; 5 Department of Paediatrics, University Children’s Hospital Queen Fabiola, Université Libre de Bruxelles, Brussels, Belgium; 6 Department of Paediatrics, San Paolo Hospital, University of Milan, Milan, Italy

**Keywords:** Sleep quantity, Child Behaviour Checklist, Emotional health, Behavioural problems, Schoolchildren

## Abstract

There is growing evidence that insufficient sleep has negative effects on the mental health of children. The aim of this study is to examine the associations between device-measured sleep duration and internalizing and externalizing problems in 8-year-old children. The study is a secondary analysis of data from the Childhood Obesity Project conducted in five European countries. Nocturnal sleep duration was measured with the SenseWear™ Armband 2. Parents rated their child’s internalizing and externalizing problems on the Child Behaviour Checklist. Behaviour scores were dichotomized at the 90th percentile based on sex- and country-specific *z*-scores. Logistic regression models were applied to test the associations between sleep duration and behaviour. Data were available for 406 8-year-old children. The average sleep duration was 9.25 h per night (SD: 0.67) with 1464 nights measured in total. The sleep duration recommendation of the American Academy of Sleep Medicine for school-aged children (9–12 h) was met by 66.7% of children. One hour of additional sleep per night significantly reduced the risk of having internalizing problems (adjusted OR = 0.51; 95% CI 0.29–0.91). Children who adhered to the sleep duration recommendation had a lower risk for internalizing problems (adjusted OR = 0.45; 95% CI 0.21–0.99). Sleep duration and externalizing problems showed no significant association. Longer sleep duration was associated with a reduced risk of having internalizing problems but not externalizing problems. Results highlight that it is important to ensure adequate sleep duration throughout primary-school years for the optimal emotional health of children. Trial registration number: NCT00338689. Registered: June 19, 2006.

**Supplementary Information:**

The online version contains supplementary material available at 10.1007/s00787-021-01731-8.

## Background

Up to 20% of children and adolescents worldwide experience mental health problems [1, 2]. It represents one of the major public health challenges in the current century [3]. Internalizing problems, like anxiety and depression, and externalizing problems, like hyperactivity, inattention or aggression, have their onset often in childhood or adolescents with tracks into adulthood [4, 5]. Therefore, it is necessary to identify potential risk factors to develop effective intervention strategies. The literature shows that health-related factors like smoking and alcohol in pregnancy or maternal overweight [6], psychosocial stressors like depression and violence [6, 7] and socio-economic factors like poverty and low education [8] can have an effect on internalizing and externalizing problems in children. Sleep might be an additional modifiable stressor that needs to be considered [9, 10].

Sleep is essential for children’s healthy development and contributes to normal mental functioning and health. Insufficient sleep is associated with negative effects on learning, memory, concentration and school performance in children [10]. Chaput et al. [11] reported in a systematic review that longer sleep duration is associated with better emotional regulation and higher quality of life in 5- to 17-year-old children and adolescents. A recent review on sleep and its relation to behaviour in preschool children demonstrated that a higher quantity of sleep is associated with better behavioural and cognitive outcomes [12]. Astill et al. [13] conducted a meta-analysis showing small but significant associations of shorter sleep duration with poorer cognition and problem behaviour in healthy school-aged children between the ages of 5 and 12 years.

However, researchers concluded that the available evidence of these studies is mainly based on parent- or self-reported sleep duration whereas more objective sleep measurement methods in children are rarely used [11, 12]. Subjective reports like questionnaires or sleep diaries often overestimate the actual sleep duration and are susceptible to reporting bias compared to device-based measurement methods like accelerometer or polysomnography [14]. Device-measured sleep is mainly used in experimental studies which have consistently shown effects of decreased sleep duration on emotional regulation, affective responses and moodiness in children and adolescents [15–17]. Only a few observational studies in children looked at associations of device-measured sleep duration and problem behaviour. They reported associations of short sleep duration with internalizing and externalizing problems as well as hyperactivity/impulsivity symptoms in school-aged children [18–20].

The main objective of our study was to examine the association of night sleep duration, measured with an accelerometer, with internalizing or externalizing symptoms in 8-year-old children, assessed with the Child Behaviour Checklist, a standardized screening questionnaire on mental health [21].

## Patients and methods

### Study design and study population

The underlying study uses data from the Childhood Obesity Project (CHOP), a double-blind randomized controlled intervention trial (ClinicalTrials.gov: NCT00338689. URL: http://clinicaltrials.gov/ct2/show/NCT00338689), initiated in 2002. Across five European countries (Belgium, Germany, Poland, Italy, Spain), 1678 healthy infants were recruited during their first eight weeks of life and randomized to either receive a higher or lower protein-content formula, with a reference group of breastfed children as control. The primary aim of the trial was to investigate whether different levels of protein-content in infant formula have an effect on infant growth and later risk of obesity. Detailed information on the whole study is published elsewhere [22, 23]. For this secondary analysis we used data from the 8-year follow-up (*N* = 589). Local ethical committees approved the trial and parents and children gave their informed consent. All research was conducted in accordance with the Declaration of Helsinki.

### Sleep duration

The nocturnal sleep duration was measured with the SenseWear*™* Armband 2 (BodyMedia Inc., Pittsburgh, PA) in 8-year-old children. As intended in the study protocol, children wore the device day and night on at least three consecutive days for at least 20 h per day. For the analyses, we included all children with at least two nights of sleep measurements. The SenseWear*™* Armband is worn on the right arm over the triceps muscle and collects data in 1 min epochs through five sensors: two-axis accelerometer, heat flux, galvanic skin response, skin temperature and near body temperature [24]*.* The two-axis accelerometer measures whether the child is lying or not. The body heat sensor can detect non-wear-time and prevents from identifying non-wear-periods as sleep [25]. Studies have shown that the SenseWear*™* Armband can give good sleep estimates compared to polysomnography [25, 26].

Sensor parameters combined with anthropometric data were edited with the Professional InnerView Software 6.1 (BodyMedia Inc., Pittsburgh, PA), which uses an undisclosed algorithm to distinguish between sleep and wake periods. Due to the lack of standardization of scoring rules for the armband, data were processed guided by scoring rules used in other studies with device-based measurements [27]. In contrast to other devices, the armband measurement differentiates lying and sleeping time. Therefore, we defined the nocturnal sleep duration as time from sleep onset (first minute of at least 3 consecutive minutes scored as lying down followed by at least 1 min scored as sleeping) to sleep offset (last minute of at least 5 consecutive minutes scored as lying). We applied the scoring rules to timeframes from 6 pm to 11 am as we could not see regular sleep duration periods during daytime.

We categorized the children in two sleep groups based on age-appropriate recommendations of the American Academy of Sleep Medicine (AASM) (9–12 h sleep per night: yes/no) [28]. Based on the armband-wear-dates the season of measurement was defined (winter [Dec–Feb], spring [Mar–May], summer [June–Aug], autumn [Sept–Nov]).

### Internalizing and externalizing problems

At the 8-year follow-up parents rated their child’s behaviour on the Achenbachs’ Child Behavior Checklist (CBCL 6/18). The CBCL is a widely used, standardized questionnaire with 113 symptom items assessed on a three-point Likert scale (‘not true = 0’, ‘somewhat or sometimes true = 1’, ‘very true or often true = 2’) [21]. From the responses, eight subscales (anxious/depressed, withdrawn/depressed, somatic complaints, rule-breaking behaviour, aggressive behaviour, thought problems, attention problems, social problems) and three broadband scales (internalizing, externalizing, total) are evaluated. The internalizing score, consists of the sum of the three subscales anxious/depressed, withdrawn/depressed and somatic complaints. The externalizing score, includes the subscales rule-breaking and aggressive behaviour. The CBCL has strong internal consistency ranging from 0.78 to 0.97 and a good test–retest reliability (*r* = 0.90) [29].

CBCL scores were *z*-standardized by sex and country to get each child’s standing relative to other children of the same sex and country in the sample [30]. All CBCL scales were positively skewed, which is typical for problem behaviour scores in a general population where most of the children have relatively low scores. Therefore, to define high internalizing and externalizing problems, we decided to dichotomize the CBCL scales at the 90th percentile of the calculated *z*-scores (cut-offs: internalizing problems: 1.53, externalizing problems: 1.32) following other studies [31, 32].

### Covariates

Additional to gender and country, various parental background information were collected at study entry. The highest education level reached by one of the parents was assessed according to the International Standard Classification of Education 1997 levels and defined as low (level 0–2), middle (level 3–4) or high (level 5–6) [33]. The mother reported her smoking status during pregnancy (yes/no) and her age at the child’s birth (years). During the 8-year follow-up the current mental health status of the mother, or in some cases of the father was measured with the General Health Questionnaire (GHQ-12) and *z*-standardized by country and respondent [34]. A higher GHQ-12 score represents poorer mental health.

### Statistical analyses

Night sleep duration was presented in decimal hours. Sex- and country-specific CBCL *z*-scores were calculated by subtracting the individual score from the corresponding country- and sex-specific mean and dividing it by the corresponding country- and sex-specific standard deviation. Differences in problem behaviour scores and nocturnal sleep duration were assessed by *t*-test and ANOVA. We applied logistic regression models to test the associations between night sleep duration and internalizing (low/high) or externalizing (low/high) problems at 8 years of age. The main predictor of the models was nocturnal sleep duration analysed as continuous (hours) and categorical data (adherence to AASM-recommendation: yes/no). Sex and country were regarded as fixed covariates for adjustment in the base models. Additional covariates for the adjusted models were selected based on a *p*-value < 0.25 in bivariate analyses (Online Resource Table 1) and the literature: highest education level reached by one of the parents, smoking in pregnancy, mother’s age at child’s birth and GHQ-12 *z*-score [6–8, 35].

Excluding children with only two nights of sleep measurements from logistic regression models, we performed a sensitivity analysis with children who had at least three nights of sleep recording as intended in the study protocol. In an additional sensitivity analysis we did not adjust for sex and country in the logistic regression models as they are already incorporated in the CBCL *z*-scores.

Statistical significance was assumed at a maximum error probability of 0.05. Statistical analysis was carried out with SPSS (IBM SPSS Statistics 26).

## Results

**Fig. 1:**
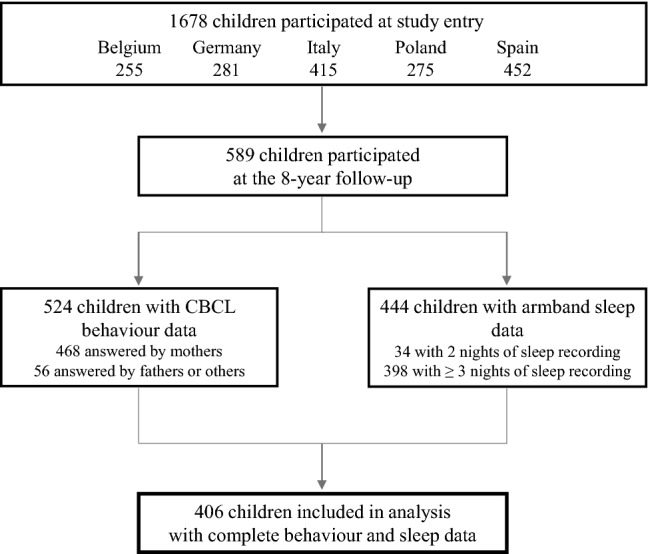
Number of participating children and available data

At the 8-year follow-up 589 children participated. CBCL data were available for 524 children (89.0%). The participation rate in armband measurement was 75.4% (444 children). CBCL scores of children who took part in the armband measurement did not differ from those who did not participate. Complete data on CBCL and sleep measurements were available for 416 children; further ten children were excluded from analysis with only one night of sleep recording. Thus, 406 children remained for the final analysis (Fig. 1). In 39 cases, fathers instead of mothers filled in the CBCL, but as there were no detectable differences of scores between fathers or mothers, we analysed them together.

Table 1 shows the characteristics of the study children and their parents. About one-third of the participating children were from Spain. Most of the parents had a middle or high level of education. Every tenth child was categorized to have high internalizing or externalizing problems as defined by decile and 27% of these children had both internalizing and externalizing problems.

**Table 1:** Characteristics of participating children and their parents

	*N* = 406^a^
Age in years, mean (SD)	7.93 (0.08)
Girls, *n* (%)	215 (53.0)
Country, *n* (%)	
Belgium	42 (10.3)
Germany	57 (14.0)
Poland	93 (22.9)
Italy	78 (19.2)
Spain	136 (33.5)
Highest education level reached by one of the parents, *n* (%)	
High	167 (41.2)
Middle	199 (49.6)
Low	35 (8.7)
Mother’s age at child’s birth in years, mean (SD)	31.09 (4.68)
Smoking in pregnancy, *n* (%)	111 (27.4)
GHQ-12 *z*-score, mean (SD)	0.00 (1.01)
Nocturnal sleep duration in decimal hours, mean (SD)	9.25 (0.67)
Adherence to AASM^b^ recommendation, *n* (%)	271 (66.7)
Season of sleep duration measurement, *n* (%)^c^	
Winter	132 (32.5)
Spring	120 (29.6)
Summer	57 (14.0)
Autumn	97 (23.9)
Number of nights measured per child, mean (SD)	3.61 (0.67)
CBCL results, *n* (%)	
High internalizing problems	40 (9.9)
High externalizing problems	40 (9.9)

*AASM* American Academy of Sleep Medicine, *CBCL* Child Behaviour Checklist, *GHQ* General Health Questionnaire, *SD* standard deviation

^a^Missings: education parents = 5; mother’s age = 1; smoking in pregnancy = 1; GHQ-12 = 24

^b^9–12 h sleep per night

^c^Meteorological classification of season: winter = December, January, February; spring = March, April, May; summer = June, July, August; autumn = September, October, November

Overall, we measured sleep duration for 1464 nights ranging from 2 to 5 nights per child. Children slept on average 9.25 h per night (SD: 0.67). We could not observe significant differences in night sleep duration due to day of sleep measurement (weekday or weekend day). Sixty-seven percent of children slept on average within the recommended range of 9–12 h per night. Boys had significantly (*p* < 0.001) shorter night sleep durations (about 15 min less) than girls (Table 2). Sleep duration differed between countries, with children from Belgium and Germany showing a longer nocturnal sleep duration than children from Italy and Spain (Table 2). There was no significant difference in sleep duration measurements based on seasons.

**Table 2:** Nocturnal sleep duration of participating children by sex, country and season of sleep duration measurement

	Nocturnal sleep duration in decimal hours	
	mean (SD)	*p* value
Sex		
Boys	9.11 (0.65)	**< 0.001**
Girls	9.36 (0.66)	
Country		
Belgium	9.74 (0.69)	**< 0.001**
Germany	9.51 (0.63)	
Poland	9.19 (0.55)	
Italy	9.11 (0.67)	
Spain	9.10 (0.65)	
Season^a^		
Winter	9.30 (0.61)	0.63
Spring	9.19 (0.67)	
Summer	9.21 (0.83)	
Autumn	9.26 (0.63)	

Significant *p* values are marked in bold

*SD* standard deviation

^a^Meteorological classification of season: winter = December, January, February; spring = March, April, May; summer = June, July, August; autumn = September, October, November

Table 3 shows the associations of night sleep duration (continuous in hours and dichotomized) with internalizing and externalizing problems (low/high). An increase of 1 h sleep duration per night was significantly related to a lower risk of having internalizing problems (adjusted OR = 0.51; 95% CI 0.29, 0.91). Further, children who adhere to the recommendation of the AASM had a lower risk for internalizing problems (adjusted OR = 0.45; 95% CI 0.21, 0.99). Base models delivered similar results (Online Resource Table 2). A poorer mental health status of the mother or father (GHQ-12 *z*-score) was significantly associated with a higher risk of internalizing problems of the child in both adjusted models (OR = 1.63; 95% CI 1.23, 2.17; OR = 1.64; 95% CI 1.24, 2.18), while none of the other covariates showed a significant association with internalizing problems.

**Table 3:** Adjusted associations between nocturnal sleep duration and internalizing and externalizing problems of children at 8 years of age

*N* = 376	High internalizing problems^a^			High externalizing problems^a^		
	OR [95% CI]	*p* value	*R*^2^	OR [95% CI]	*p* value	*R*^2^
Nocturnal sleep duration in h	0.51 [0.29, 0.91]	**0.02**	13.7	0.77 [0.44, 1.35]	0.36	13.6
Adherence to AASM recommendation^b^	0.45 [0.21, 0.99]	**0.046**	13.0	0.53 [0.25, 1.16]	0.11	14.5

Significant *p* values are marked in bold

All models were adjusted for sex, country, highest level of education reached by one of the parents, mother’s age at child’s birth, smoking in pregnancy and mother/father GHQ-12 *z*-score

*AASM* American Academy of Sleep Medicine, *OR* Odds Ratio, *R*^2^ Nagelkerkes *R*^2^, *95% CI* 95% confidence interval

^a^Low internalizing and externalizing problems were defined as scores below the 90th percentile on the internalizing and externalizing scale of the CBCL and high internalizing and externalizing problems as scores at the 90th percentile or higher on the internalizing and externalizing scale of the CBCL

^b^9–12 h sleep per night

Nocturnal sleep duration in hours and externalizing problems showed no significant association (adjusted OR = 0.77; 95% CI 0.44–1.35). Further, there was no association between adherence to AASM recommendation and externalizing problems (adjusted OR = 0.53; 95% CI 0.25–1.16). Base models obtained similar results (Online Resource Table 2). A poorer mental health status of the mother or father was significantly associated with a higher risk of externalizing problems of the child in both adjusted models (OR = 1.56; 95% CI 1.19, 2.05; OR = 1.55; 95% CI 1.18, 2.03). Furthermore, the mother’s age at child’s birth showed a significant association with externalizing problems (adjusted OR = 0.91; 95% CI 0.83, 0.99; adjusted OR = 0.91; 95% CI 0.83, 0.99). None of the other covariates were significantly associated with externalizing problems.

Sensitivity analysis on the sample with three or more nights of sleep recording and without adjusting for sex and country delivered similar results (Online Resource Tables 3 and 4). Online Resource Table 5 shows the associations of night sleep duration with the subscales of internalizing and externalizing problems. There was no significant association in a specific subscale.

## Discussion

The present study investigated device-measured nocturnal sleep duration and its association with internalizing and externalizing problems in 8-year-old children from five European countries. Besides sex and country differences in sleep durations, we observed that an increase of 1 h sleep duration per night or the adherence to the AASM recommendation was significantly related to a lower risk of having internalizing problems even when controlling for other variables, while sleep duration and externalizing problems were not associated.

Some observational and experimental studies looked at device-measured sleep and different spectrums of problem behaviour in children. Most of the observational studies have a cross-sectional design. In the study of Nixon et al. [18] sleep of 519 7-year-old children was measured by actigraphs and dichotomized into less than 9 h and more than 9 h sleep per night. Similar to our results, less than 9 h sleep was significantly associated with higher emotional lability scores compared to children sleeping more than 9 h per night. Externalizing symptoms like attention deficit or hyperactivity disorder scores did not differ with sleep duration. Emotional lability scores and externalizing symptoms were measured by the Conners Rating Scale Parent Form. However, the results were based on sleep duration of just one night measurement. Another actigraph-study observed that a decreased sleep duration was not associated with parent-reported CBCL externalizing symptoms in 49 school-aged children but with teacher-reported externalizing symptoms measured by the Teacher’s Report Form [19]. The results suggest that externalizing problems seem to be more evident in the school environment than at home. This could be a reason why we did not find an association between sleep and parent-reported externalizing problems.

Paavonen et al. [20] showed that 280 8-year-old children with less than 7.7 h of sleep per night, measured by actigraphs, had an increased risk for behavioural problems like hyperactivity/impulsivity symptoms using maternal ratings of the ADHD Rating Scale IV. Differences to our results can be due to other cut-offs to define short sleep (7.7 vs. 9 h in our study) and the focus on attention problems and hyperactivity symptoms rather than on aggressive and rule-breaking behaviour. As attention problems and hyperactivity are not part of the CBCL externalizing score, this spectrum of behavioural problems was not included in our analysis.

Longitudinal studies with objective sleep measurement methods are relatively rare in children and have suggested that reduced sleep is associated with an increased risk of future occurrence of emotional and externalizing symptoms [36, 37].

A few studies in children have examined experimentally induced sleep deprivation. Vriend et al. [15] showed in 8- to 12-year-old children that going to bed 1 h later for four nights relative to the typical bedtime had significant consequences on emotion regulation and positive affective responses measured by the parent-reported Emotion Questionnaire and an Affective Response Task. Another study in 50 adolescents aged 14–17 years reported that sleep restriction to 6.5 h in bed per night for five nights resulted in poorer emotional regulation and more feelings of anxiety, anger and tension (self-reports on Profile of Moods States) compared to the healthy sleep duration group (10 h in bed per night for five nights) [16]. Gruber et al. [17] observed that a restriction of sleep over five nights in 7- to 11-year-old children had negative effects on emotionality and moodiness in school, measured by the Conners’ Global Index—Teachers, compared to children with extended sleep. These findings imply that even a modest sleep restriction of a realistic amount of sleep over a few nights can weaken the ability to regulate emotions, which can lead to problem behaviour in children. This emphasizes the importance of perceiving sleep as a potentially modifiable factor in children’s emotional health.

In the literature, several hypotheses try to explain how sleep and behaviour might be related. The overnight therapy hypothesis for example proposed that sleep provides a timeframe for resetting the neuronal systems [38]. One experimental study in adults showed that severe sleep deprivation leads to a more intense amygdala response to negative emotional stimuli compared to individuals who were not sleep-deprived [39]. As this system is involved in the affect regulation and processing of emotions, insufficient sleep can have negative impacts on emotional regulation and mood. The applicability of this hypothesis for children needs further clarification.

Our analysis showed sex and country-specific differences in sleep duration. Girls slept on average 15 min longer than boys, a finding which is consistent with previous studies in school-aged children [40, 41]. The cause for the observed sex difference is still unclear, but there is some support that girls are more sensitive to their sleep requirements and that parenting styles or socio-cultural effects may play a role [40, 41].

Nocturnal sleep duration differed between countries, with children from middle Europe (Germany, Belgium) showing a longer sleep duration than children in southern (Italy, Spain) or eastern (Poland) countries. This is in line with results from the IDEFICS study that reported significant differences between sleep durations in eight European countries, with children from northern countries sleeping longer than children in middle or southern Europe [42]. One reason could be cultural differences in sleep habits [42]. Norms and expectations regarding normal and problematic sleep of children may differ between countries and can lead to various bedtime routines. In addition, social demands like school starting times can vary in countries and regulate the sleep of school-children differently [43].

### Strengths and limitations

One strength of the study is the multicentre design with participants from metropolitan areas of five European countries. This makes it possible to generalize the results to other European children with similar demographic backgrounds and living conditions.

Another strength includes the device-measured nocturnal sleep duration. The gold standard for sleep measurements is laboratory measurements like polysomnography, which are not applicable in larger epidemiological studies. The SenseWear*™* Armband is easy to handle and does not disturb the usual sleep habits of children [25, 26]. There is a paucity of validation studies for the SenseWear*™* Armband and its use for sleep in normative samples of children. Two studies have shown that the armband was less accurate on an individual level compared to polysomnography measurements which prevents the armband from being used as a clinical tool [25, 26]. Furthermore, due to the lack of standardization of scoring rules for the armband, we based our sleep definition on scoring rules used in actigraph-studies even if we used an arm placement and not a wrist or waist placement [27].

It is difficult to directly compare the results of accelerometer-studies with our study due to various devices used and a different number of days measured. One limitation of our study is the relatively short observation time from 2 to 5 nights. The AASM recommends for actigraph studies a recording time for a minimum of 72 h and other studies reported four to seven nights [44, 45]. However, our measurements provided reasonable values for night sleep duration and are comparable to a meta-analysis of actigraphically-measured sleep in 6- to 8-year-old children (range of the pooled mean sleep duration: 8.53–9.43 h) [46].

Furthermore, the CBCL assessment was based on parent-reported data and could be affected by socially desirable answers despite guaranteed anonymity. Parents’ expectations regarding a normal or problematic behaviour of children could have been affected the scores. Additionally, the selection of externalizing problem items in the CBCL does not cover attention problems and hyperactivity. Nevertheless, the CBCL is a well-standardized screening tool fitting exactly for the age group and is designed to be filled by parents.

A further limitation is the cross-sectional design of the study that does not allow an interpretation of longitudinal relationships.

## Conclusion

In a cross-sectional multicentre study in 8-year-old European children, each additional hour of nocturnal sleep duration and the adherence to the AASM recommendation reduced the risk of having internalizing problems. Externalizing problems were not associated with night sleep duration.

Adequate sleep duration throughout primary-school years is important for children’s optimal emotional health. Pediatricians should consider sleep as a potential risk factor for internalizing problems in children. Further research on longitudinal associations is needed to determine whether short sleep is a cause or consequence of problem behaviour.

## Supplementary Information

Below is the link to the electronic supplementary material.

Supplementary file1 (DOCX 30 KB)

## Data Availability

The authors support sharing data with other researchers for legitimate research purposes. However, the study is still ongoing and data cannot yet be anonymized as we currently plan a further follow-up. Therefore, according to the General Data Protection Regulation and the institution’s data protection rules individual study participant data cannot be put in the public domain but can only be shared after establishing a written data sharing agreement ensuring that collaborating researchers do not violate privacy regulations and are in keeping with informed consent that is provided by study participants. Written requests to access the data may be submitted to: office.koletzko@med.uni-muenchen.de.

## Abbreviations

AASM: American Academy of Sleep Medicine
CBCL: Child Behaviour Checklist
CHOP: Childhood Obesity Project
CI: Confidence interval
GHQ: General Health Questionnaire
OR: Odds ratio
SD: Standard deviation

## Code availability

Statistical analysis was carried out with SPSS (IBM SPSS Statistics 26).
