# Supplementary material for: Sleep duration and problem behaviour in 8-year-old children in the Childhood Obesity Project
Source: Eur Child Adolesc Psychiatry. 2021 Feb 24;31(3):519–27. doi: 10.1007/s00787-021-01731-8 (PMC8940797; doi:10.1007/s00787-021-01731-8)
Supplement: Supplementary file 1 — Supplementary file1 (DOCX 30 KB) [file 787_2021_1731_MOESM1_ESM.docx]

## Online Resource

**Article title:** Sleep duration and problem behaviour in 8-year-old children in the Childhood Obesity Project

**Journal name:** European Child & Adolescent Psychiatry

**Author names:** Kathrin Guerlich, Dariusz Gruszfeld, Justyna Czech-Kowalska, Natàlia Ferré, Ricardo Closa-Monasterolo, Françoise Martin, Pascale Poncelet, Elvira Verduci, Berthold Koletzko, Veit Grote

**Corresponding author:** Veit Grote, Division of Metabolic and Nutritional Medicine, Department of Pediatrics, Dr. von Hauner Children’s Hospital, LMU University Hospital Munich, Germany, E-mail: [veit.grote@med.uni-muenchen.de](mailto:veit.grote@med.uni-muenchen.de)

**Table 1:** Bivariate associations between internalizing and externalizing problems, sleep and covariates

| N=406^a^ | **Internalizing problems** | | **Externalizing problems** | |
| --- | --- | --- | --- | --- |
|  | OR [95% CI] | p-value | OR [95% CI] | p-value |
| **Sleep** |  |  |  |  |
| **Sleep duration in h** | 0.47 [0.29-0.76] | 0.002 | 0.77 [0.47-1.25] | 0.29 |
| **Adherence to ASSM recommendation (Yes)**^b^ | 0.46 [0.24-0.89] | 0.02 | 0.64 [0.33-1.25] | 0.19 |
| **Study country** |  |  |  |  |
| Belgium | 0.73 [0.20-2.69] | 0.63 | 1.54 [0.50-4.70] | 0.45 |
| Germany | 0.91 [0.31-2.68] | 0.86 | 1.09 [0.36-3.30] | 0.88 |
| Poland | 1.14 [0.48-2.72] | 0.77 | 1.37 [0.56-3.37] | 0.49 |
| Italy | 1.23 [0.50-3.03] | 0.65 | 1.48 [0.59-3.75] | 0.41 |
| Spain | Reference | - | Reference | - |
| **Parental characteristics** |  |  |  |  |
| **Highest education level reached by one of the parents** |  |  |  |  |
| No/low | 1.53 [0.47-5.00] | 0.48 | 2.26 [0.80-6.37] | 0.12 |
| Middle | 1.47 [0.72-3.02] | 0.29 | 1.15 [0.56-2.38] | 0.70 |
| High | Reference | - | Reference | - |
| **GHQ-12 z-score** | 1.62 [1.25-2.11] | <0.001 | 1.59 [1.23-2.06] | <0.001 |
| **Mother’s age at child’s birth (years)** | 0.94 [0.87-1.00] | 0.06 | 0.94 [0.87-1.00] | 0.06 |
| **Smoking in pregnancy (yes)** | 1.37 [0.68-2.77] | 0.38 | 0.55 [0.24-1.29] | 0.17 |
| **Child characteristics** |  |  |  |  |
| **Sex (Boy)** | 0.82 [0.42-1.58] | 0.55 | 1.14 [0.59-2.19] | 0.69 |

Abbreviations: AASM: American Academy of Sleep Medicine, GHQ: General Health Questionnaire, OR: Odds Ratio, 95% CI: 95% confidence interval

^a^ Missings: education mother=2; education father=4; mother’s age=1; smoking in pregnancy=1; GHQ-12 z-score=24

^b^ 9-12 hours sleep per night

**Table 2:** Base models: Associations between sleep duration and internalizing and externalizing problems

| n=406 | High internalizing problems^a^ | | | High externalizing problems^a^ | | |
| --- | --- | --- | --- | --- | --- | --- |
|  | OR [95% CI] | p-value | R^2^ | OR [95% CI] | p-value | R^2^ |
| Sleep duration in h | 0.43 [0.26, 0.72] | 0.001 | 5.9 | 0.73 [0.44, 1.23] | 0.24 | 1.4 |
| Adherence to AASM recommendation^b^ | 0.44 [0.22, 0.87] | 0.02 | 3.5 | 0.62 [0.31, 1.23] | 0.17 | 1.6 |

Note: All models were adjusted for sex and country.
Abbreviations: AASM: American Academy of Sleep Medicine, OR: Odds Ratio, R^2^: Nagelkerkes R^2^, 95% CI: 95% confidence interval

^a^ Low internalizing and externalizing problems were defined as scores below the 90th percentile on the internalizing and externalizing scale of the CBCL and high internalizing and externalizing problems as scores at the 90th percentile or higher on the internalizing and externalizing scale of the CBCL.
^b^ 9-12 hours per night

**Table 3**: Sensitivity analysis: Adjusted associations between sleep duration and internalizing and externalizing problems, excluding children with only two nights of sleep recording

| n=348 | High internalizing problems^a^ | | | High externalizing problems^a^ | | |
| --- | --- | --- | --- | --- | --- | --- |
|  | OR [95% CI] | p-value | R^2^ | OR [95% CI] | p-value | R^2^ |
| Sleep duration in h | 0.46 [0.24, 0.87] | 0.02 | 12.8 | 0.66 [0.36, 1.23] | 0.19 | 13.2 |
| Adherence to AASM recommendation^b^ | 0.46 [0.21, 1.03] | 0.058 | 11.3 | 0.51 [0.23, 1.12] | 0.09 | 13.8 |

*Note: All models were adjusted for sex, country, highest level of education reached by one of the parents, , mother’s age at birth, smoking in pregnancy and mother/father GHQ-12 z-score.
Abbreviations: AASM: American Academy of Sleep Medicine, OR: Odds Ratio, R^2^: Nagelkerkes R^2^, 9*5% *CI: 95% confidence interval*

*^a^ Low internalizing and externalizing problems were defined as scores below the 90th percentile on the internalizing and externalizing scale of the CBCL and high internalizing and externalizing problems as scores at the 90th percentile or higher on the internalizing and externalizing scale of the CBCL.*
*^b^ 9-12 hours per night*

**Table 4**: Sensitivity analysis: Adjusted associations between sleep duration and internalizing and externalizing problems, not adjusting for sex and country

| n=376 | High internalizing problems^a^ | | | High externalizing problems^a^ | | |
| --- | --- | --- | --- | --- | --- | --- |
|  | OR [95% CI] | p-value | R^2^ | OR [95% CI] | p-value | R^2^ |
| Sleep duration in h | 0.51 [0.30, 0.87] | 0.01 | 12.5 | 0.77 [0.46, 1.31] | 0.34 | 12.7 |
| Adherence to AASM recommendation^b^ | 0.45 [0.21, 0.94] | 0.03 | 11.6 | 0.54 [0.26, 1.13] | 0.10 | 13.6 |

*Note: All models were adjusted forhighest level of education reached by one of the parents, , mother’s age at birth, smoking in pregnancy and mother/father GHQ-12 z-score.
Abbreviations: AASM: American Academy of Sleep Medicine, OR: Odds Ratio, R^2^: Nagelkerkes R^2^, 9*5% *CI: 95% confidence interval*

*^a^ Low internalizing and externalizing problems were defined as scores below the 90th percentile on the internalizing and externalizing scale of the CBCL and high internalizing and externalizing problems as scores at the 90th percentile or higher on the internalizing and externalizing scale of the CBCL.*
*^b^ 9-12 hours per night*

**Table 5**: Adjusted associations between sleep duration and subscales of internalizing and externalizing problems

| n=376 | Internalizing problems | | | | | | | | | | | |  | | | Externalizing problems | | | | | |  | |
| --- | --- | --- | --- | --- | --- | --- | --- | --- | --- | --- | --- | --- | --- | --- | --- | --- | --- | --- | --- | --- | --- | --- | --- |
|  | | High  anxious/depressed problems^a^ | | | | High  withdrawn/depressed problems^a^ | | | | High  somatic complaints^a^ | | | | | High  rule-breaking behaviour^a^ | | | | | High  aggressive behaviour^a^ | | | |
|  | OR  [95% CI] | | p | R^2^ | OR  [95% CI] | | p | R^2^ | OR  [95% CI] | | p | R^2^ | | OR  [95% CI] | | | p | R^2^ | OR  [95% CI] | | p | | R^2^ |
| Sleep duration in h | 0.65  [0.37, 1.16] | | 0.15 | 12.9 | 0.67  [0.38, 1.21] | | 0.18 | 4.0 | 0.71  [0.41, 1.23] | | 0.22 | 12.2 | | 1.18  [0.65, 2.13] | | | 0.59 | 4.6 | 0.76  [0.43, 1.34] | | 0.34 | | 18.7 |
| Adherence to AASM recommendation^b^ | 0.53  [0.24, 1.16] | | 0.11 | 13.1 | 0.65  [0.30, 1.39] | | 0.26 | 3.7 | 0.60  [0.28, 1.32] | | 0.21 | 12.2 | | 1.17  [0.53, 2.59] | | | 0.70 | 4.5 | 0.48  [0.22, 1.04] | | 0.06 | | 20.0 |

*Note: All models were adjusted for sex, country, highest level of education reached by one of the parents, , mother’s age at birth, smoking in pregnancy and mother/father GHQ-12 z-score.
Abbreviations: AASM: American Academy of Sleep Medicine, OR: Odds Ratio, R^2^: Nagelkerkes R^2^, 9*5% *CI: 95% confidence interval*

*^a^ Low problems were defined as scores below the 90th percentile on the anxious/depressed, withdrawn/depressed, somatic complaints, rule-breaking behaviour and aggressive behaviour scale of the CBCL and high problems as scores at the 90th percentile or higher on the anxious/depressed, withdrawn/depressed, somatic complaints, rule-breaking behaviour and aggressive behaviour problem scale of the CBCL.
^b^ 9-12 hours per night*
